# Disseminated Cryptococcosis in an HIV-Negative Patient With Liver Cirrhosis and Asplenia: A Rare but Dreadful Disease

**DOI:** 10.7759/cureus.37243

**Published:** 2023-04-07

**Authors:** Venu Madhav Chippa, Swetha Chenna, Rahul Gujarathi, Narsimha Candula

**Affiliations:** 1 Internal Medicine, St. Vincent Medical Center, Evansville, USA; 2 Internal Medicine, Indiana University, Indianapolis, USA; 3 Hospital Medicine, University of Florida Health, Jacksonville, USA

**Keywords:** multiorgan system failure, hiv negative, asplenia, liver cirrhosis, disseminated cryptococcal infection

## Abstract

Cryptococcosis (cryptococcal infection) is a severe life-threatening fungal infection. It is seen worldwide, specifically in immunocompromised, mainly in human immunodeficiency virus/acquired immunodeficiency syndrome (HIV/AIDS)-infected individuals. Cryptococcal infection can present with meningitis, pneumonia, peritonitis, disseminated cryptococcosis, and cryptococcal fungemia. Here, we report the case of an HIV-negative Caucasian male in his early 50s with liver cirrhosis and asplenia who presented to our hospital with bilateral foot cellulitis and pneumonia. He was eventually diagnosed with disseminated cryptococcosis. Even with appropriate treatment, he developed multiorgan failure and finally expired. The disseminated cryptococcal infection has a very high mortality rate in patients with liver cirrhosis and asplenia. Liver cirrhosis is an independent risk factor, and asplenia is a comorbid condition for cryptococcal infection in HIV-negative patients. Healthcare providers should have a high suspicion of cryptococcosis in these patients. Early testing with cryptococcal antigen assay and initiation of an appropriate antimicrobial regimen can help minimize bad outcomes.

## Introduction

*Cryptococcus neoformans* (CN) and *Cryptococcus gattii* (CG) are ubiquitous invasive fungi transmitted through the inhalation of microscopic spores and cause cryptococcosis [1]. Despite the lung being the site from where cryptococcus enters the body, meningoencephalitis is the most common clinical manifestation. CN infections are more common than CG infections and can cause meningitis, peritonitis, pneumonia, urinary tract infection, cellulitis, osteomyelitis, and even disseminated infection, causing multiorgan failure [2]. The most common symptoms in patients with cryptococcal infection are fever, nausea, vomiting, headache, neck stiffness, abdominal pain, difficulty breathing, and dizziness [1,2].

Worldwide, over a million cases of cryptococcosis are reported each year, with approximately 625,000 deaths [1]. The estimated incidence of cryptococcosis in the United States is about 0.4-1.3 cases per 100,000. In people with acquired immunodeficiency syndrome (AIDS), the incidence is 2-7 cases per 100,000, with a case fatality rate of about 12% [2]. The global incidence of cryptococcal infections in human immunodeficiency virus (HIV) has declined drastically over the last two decades owing to advances in antiretroviral therapy [2].

HIV/AIDS, decompensated liver disease, cell-mediated immunosuppressive regimen without calcineurin inhibitors, long-term steroid use, and autoimmune diseases are independent risk factors for invasive cryptococcosis [3]. Decompensated cirrhosis patients with Child-Pugh class B and C are more likely to have extrapulmonary cryptococcosis which is associated with increased mortality [4]. Invasive procedures, long-term steroid use, antibiotics use, malnutrition, asplenia, active cancer, diabetes mellitus, and solid organ transplantation are comorbid conditions for cryptococcosis in HIV-negative patients [5]. With increased awareness and testing, cryptococcosis is increasingly reported in patients with cirrhosis, accounting for 6-21% of all systemic fungal infections with a mortality rate of up to 76% [6,7].

Many cases of disseminated cryptococcal infection with liver cirrhosis are reported in medical literature and only two cases with asplenia. The disseminated cryptococcal infection was not previously reported in patients with combined asplenia and liver cirrhosis. This case report highlights the increased probability of invasive cryptococcal infection in asplenia with liver cirrhosis. We encourage healthcare providers to identify this dreadful infection early to improve patient outcomes.

## Case presentation

A Caucasian male in his early 50s was sent from his podiatrist’s office to the emergency room for non-healing bilateral foot ulceration, cellulitis, productive cough, and fever. His past medical history was significant for alcoholic liver cirrhosis, no ascites or paracentesis, and non-healing bilateral chronic foot ulcers requiring multiple rounds of oral and intravenous (IV) antibiotics. He was currently not on any medications. His previous wound cultures grew methicillin-resistant *Staphylococcus aureus*. He had quit alcohol four years ago and had never smoked.

On examination, his temperature was 38.9°C (102.1°F), pulse rate was 117 beats/minute, respiratory rate was 20 breaths/minute, and blood pressure was 146/85 mmHg. He appeared in mild respiratory distress. Lungs were clear to auscultation with slightly decreased breath sounds in the left base and regular S1-S2 with tachycardia. His abdomen was non-distended and non-tender with normal bowel sounds. Bilateral extremities skin showed multiple superficial wounds on the dorsum of the feet, with erythema and purulent drainage.

A basic workup in the emergency room showed an elevated white blood cell count (WBC) (18,600/µL) and lactic acid (5 mmol/L). Other laboratory values were normal, including liver function tests, creatinine, electrolytes, hemoglobin, platelets, coagulation panel, and urinalysis. He was admitted to the medical floor. X-rays of both feet showed no osteomyelitis, and a chest X-ray showed a small left lower lobe consolidation and a trace left pleural effusion.

He was admitted for sepsis from left-sided pneumonia and possible recurrent cellulitis of bilateral foot ulcerations secondary to chronic venous stasis. Blood culture and wound culture were obtained, and he received goal-directed IV fluid resuscitation for sepsis and was started on IV vancomycin and piperacillin-tazobactam.

**Figure 1:**
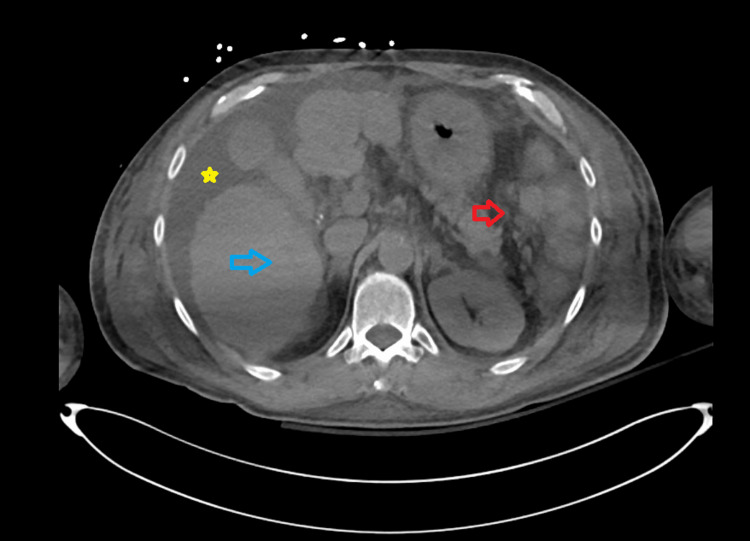
CT of the abdomen without contrast. CT of the abdomen without contrast showing cirrhosis (blue arrow), ascites (yellow star), and absent spleen (red arrow).

The following day he complained of worsening shortness of breath, requiring high-flow oxygen and a transfer to the intensive care unit (ICU). To better identify the cause of his symptoms, a computerized tomogram (CT) of the chest, abdomen, and pelvis without IV contrast was performed. CT showed bilateral lung consolidation (more on the left), with moderate left and small right pleural effusions and slight ascites (Figure 1).

A CT of the bilateral lower extremities without contrast showed subcutaneous edema suggestive of cellulitis. He underwent emergent left-sided thoracentesis to relieve his hypoxia and respiratory distress, and pleural fluid analysis showed elevated WBC, lactate dehydrogenase, and neutrophils, as shown in Table 1.

**Table 1:** Pleural fluid analysis. RBC: red blood cell; LDH: lactic acid dehydrogenase

Characteristics	Patient’s range	Normal range
Color	Yellow	Straw color
Appearance	Cloudy	clear
RBC (/mm^3^)	<10,000	<10,000
WBC (/mm^3^)	2,229	1,000
Neutrophil (%)	78	1
Lymphocytes (%)	15	23
Monocytes (%)	4	0-1
Macrophages (%)	0	75
Mesothelial cells (%)	1	1-2
Basophils (%)	0	0-1
Eosinophils (%)	2	1
LDH (U/L)	320	No reference range established
Protein (g/dL)	2	No reference range established
Albumin (g/dL)	1	No reference range established
pH	7.5	7.6–7.64

Follow-up lactic acid was 7.8 mmol/L and 11 mmol/L. Ultrasound of the abdomen did not show any ascitic fluid amenable for paracentesis. He had a negative HIV antibody (enzyme-linked immunoassay) test and polymerase chain reaction.

On hospital day four, his mentation deteriorated. He became hypotensive and anuric and developed respiratory failure requiring intubation. Vancomycin trough levels were normal, and we thought that the ongoing sepsis might be the reason for the overall decline in the clinical situation. Admission blood cultures grew CN. We were convinced he might have disseminated cryptococcal infection and immediately started him on liposomal amphotericin B. We could not administer flucytosine because of his end-stage liver disease. Serum cryptococcal antigen measured by indirect enzyme immunoassay (EIA) was 1:32.

Follow-up blood and pleural fluid cultures grew CN. Because of fungemia and altered sensorium before intubation, a lumbar puncture was performed on day five, which showed an elevated opening pressure with significant cerebrospinal fluid (CSF) cryptococcal antigen titer, as shown in Table 2.

**Table 2:** Cerebrospinal fluid analysis. CSF: cerebrospinal fluid; WBC: white blood cell

Characteristics	Patient’s range first tap	Patient’s range second tap	Normal range
Pressure (cm of H₂O)	40	30	5–20
Appearance	Hazy	Hazy	Clear
Protein (mg/dL)	1.88	2.00	1.8–4.5
Glucose ( mg/dL)	33	35	50–80
Microscopic examination	Yeast	Yeast	None
Cell count	Lymphocytes 34%, monocytes 54%	Lymphocytes 36%, monocytes 52%	None
CSF cryptococcal antigen titer	1:640	1:640	
WBC count (cells/µL)	128	99	0–5

He underwent repeated spinal taps with a slight improvement in opening pressures, and persistent yeast forms were seen on microscopic examination. He was finally diagnosed with a disseminated cryptococcal infection, likely secondary to liver cirrhosis, asplenia, and a recent course of prolonged antibiotics. His wound culture only grew *Klebsiella *and *Pseudomonas*.

Unfortunately, his hospital course deteriorated. He developed shock and acute renal injury and required vasopressors and continuous renal replacement therapy. He developed disseminated intravascular coagulation and multiorgan failure with no meaningful outcome and progressively deteriorating health. His family decided to provide comfort care. The patient passed peacefully on day 14 of hospital admission.

## Discussion

Disseminated cryptococcosis is defined by either a positive blood culture (fungemia) or a single positive culture from at least two organ systems [8]. Cirrhotic patients have compromised immunity, making them more prone to opportunistic infections, particularly CN. Asplenic patients are more prone to encapsulated bacterial infections, but some case reports suggest the prevalence of fungal infections, specifically CN. Although the respiratory tract is the usual port of entry for cryptococcus, the gastrointestinal tract can also serve as another potential entry site in immunocompromised patients with HIV, liver cirrhosis, and a history of renal disease [9].

In a retrospective study by Chuang et al., in HIV-uninfected patients with cryptococcosis, 36% had liver cirrhosis, 33% had diabetes mellitus, and 27% had autoimmune diseases. All patients with liver cirrhosis and disseminated cryptococcosis died within the first month [10]. A retrospective study by Zhou et al. showed that increased activated partial thromboplastin time and Child-Pugh class B or C were associated with increased mortality with cryptococcosis in liver cirrhosis. The Model for End-stage Liver Disease sodium score was significant for predicting 30-day mortality, and the Child-Pugh score was more helpful in predicting 90-day mortality [11]. The risk factors for cryptococcus in our patient were a Child-Pugh score of 9 (class B), asplenia, and multiple rounds of antibiotics before this hospital admission.

The host immune response to cryptococcal infection includes cell-mediated (T cell and natural killer cell) and humoral (antibody) immunity. In cirrhosis, innate and adaptive immunity failure leads to altered intracellular signaling pathways, damaging gastrointestinal tract lymphoid tissues, and circulating immune cells. This phenomenon, called cirrhosis-associated immune dysfunction syndrome, is thought to contribute to the increased incidence of systemic fungal infections in cirrhosis [12]. The precise mechanism for increased mortality from fungal infections in asplenia in humans is not known. Still, animal models suggest the abnormal antimicrobial function of peritoneal macrophages (PM phi) affects intracellular fungal (*Candida*) destruction and increases mortality [13].

Cryptococcal infection can present as meningitis, pneumonia, peritonitis, and fungemia. Sometimes pleural effusion is also present along with pneumonia. Both pleural and ascitic fluid is usually exudative with predominant lymphocytes and culture negative for bacterial growth.

Testing for cryptococcal disease has evolved over the last few years, with particular improvements in cryptococcal latex antigen (CrAg) testing. The sensitivity of CrAg is 97.5%, and the specificity is 85-100%. A new point-of-care lateral flow immunoassay is currently being used with a sensitivity and specificity of 99% and has a rapid turnaround time of as little as 12 hours [14]. In a retrospective, observational study by Cheng et al., the most common cryptococcal infection in liver cirrhosis is meningitis, pneumonia, fungemia, and skin and bone infection [4]. Blood, CSF, pleural fluid, and peritoneal fluid cryptococcal antigen assays rapidly identify cryptococcal infection in high-risk populations. Microscopic examination and cultures are incredibly beneficial but can delay diagnosis, thus missing the opportunity for early treatment [15]. The Infectious Disease Society of America (IDSA) recommends that all patients with pulmonary cryptococcosis and fungemia get tested for cryptococcal meningitis regardless of the risk factors. In a retrospective study by Bradley et al., around 40% of patients without HIV who had pulmonary cryptococcosis had disseminated disease, including meningitis [16]. Our patient had pulmonary, meningeal, and bloodstream cryptococcal infections. All patients should get tested for HIV.

The current standard of therapy for cryptococcal meningitis and disseminated cryptococcal infection constitutes induction therapy with amphotericin B plus flucytosine for two weeks, followed by consolidation with 400 mg daily for fluconazole for eight weeks and 200 mg/day for six months as maintenance therapy (IDSA recommendation). Despite dose-limiting toxicity from amphotericin B, it is the standard treatment for disseminated cryptococcal infection as it is fungicidal. Using lipid carriers-liposomal formulation, lipid complex formulation, and a colloidal dispersion reduces the side effects of amphotericin B. Resistance to amphotericin B is extremely rare. Flucytosine should be used with amphotericin B as it is highly hepatotoxic and myelotoxic [17]. A study by Tariq et al. showed that antifungal therapy is underutilized in non-HIV immunocompromised populations [18]. A retrospective study published in July 2022 by Liu et al. showed amphotericin B, flucytosine combined with voriconazole rapidly improves clinical manifestation, decreases CSF opening pressure, clears cryptococcus in CSF in the early phase, substantially shortens the hospitalization time in non-HIV and non-transplant-associated cryptococcal meningitis [19].

Screening for cryptococcal antigen is of limited use in asymptomatic HIV-negative patients. A prospective study by Suh et al. showed that serum cryptococcal antigen positivity is very low in non-infectious hospitalized liver cirrhosis patients [20]. Further studies are needed to evaluate this recommendation. However, in people with HIV/AIDS, the CrAg test can positively detect the cryptococcal antigen in serum 22 days before symptoms of meningitis develop and helps save lives [21].

Our case demonstrates the importance of considering cryptococcal infection even in well-compensated liver cirrhosis (Child-Pugh class B) and other comorbid factors such as asplenia and prolonged antibiotics use. Our case was challenging because the patient presented with sepsis from both cellulitis and pneumonia, which later became a disseminated cryptococcal infection. Despite appropriate antimicrobial treatment, his hospital course worsened rapidly and was ultimately fatal. Unfortunately, we did not perform the serum CrAg test in our emergency room. Performing a serum CrAg test in the emergency room is helpful in either ruling in or ruling out cryptococcus infection early. It facilitates the early initiation of appropriate antifungal treatment in patients with risk factors.

## Conclusions

CN is a common fungal infection in HIV and AIDS patients. Still, this dreadful infection can also affect other immunocompromised people (liver cirrhosis, diabetes mellitus, etc.). A high index of suspicion should be kept for cryptococcal infection in high-risk individuals as it has very non-specific symptoms such as fever, nausea, headache, and dizziness. Serum, CSF, and peritoneal fluid CrAg testing should be done along with blood and CSF cultures, and CrAg tests have a rapid turnaround time. Indian Ink CSF fluid analysis can also be used but has low sensitivity. Disseminated cryptococcal infections typically have poor outcomes even with aggressive treatment, and early identification might play a role in improving outcomes.
